# Effect of probiotics at different intervention time on glycemic control in patients with type 2 diabetes mellitus: a systematic review and meta-analysis

**DOI:** 10.3389/fendo.2024.1392306

**Published:** 2024-07-24

**Authors:** Xinghui Wang, Lu Chen, Chunling Zhang, Qing Shi, Lei Zhu, Sisi Zhao, Zhiqin Luo, Yirun Long

**Affiliations:** ^1^ School of Nursing, Guizhou University of Traditional Chinese Medicine, Guiyang, Guizhou, China; ^2^ Department of Endocrinology and Metabolism, The Second Affiliated Hospital of Guizhou University of Traditional Chinese Medicine, Guiyang, Guizhou, China; ^3^ Department of Nutrition, The Second Affiliated Hospital of Guizhou University of Traditional Chinese Medicine, Guiyang, Guizhou, China

**Keywords:** type 2 diabetes mellitus, diabetes mellitus, gut microbiota, probiotics, glycemic control, intervention time

## Abstract

**Background:**

Type 2 diabetes mellitus(T2DM) is characterized by hyperglycemia. Gut microbiome adjustment plays a positive part in glucose regulation, which has become a hotspot. Probiotics have been studied for their potential to control the gut flora and to treat T2DM. However, the conclusion of its glucose-lowering effect is inconsistent based on different probiotic intervention times.

**Objectives:**

To comprehensively evaluate how various probiotic intervention times affect glycemic control in people with T2DM.

**Methods:**

We retrieved PubMed, Embase, Web of Science, and Cochrane Library on randomized controlled trials(RCTs)regarding the impact of probiotics on glycemic control in patients with T2DM from the inception to November 16, 2023. Separately, two researchers conducted a literature analysis, data extraction, and bias risk assessment of the involved studies. We followed the PRISMA guidelines, used RevMan 5.4 software for meta-analysis, and assessed the risk of bias by applying the Cochrane Handbook for Systematic Reviews 5.1.0.

**Results:**

We included eight RCTs with 507 patients. Meta-analysis revealed that the use of probiotics might considerably reduce levels of glycosylated hemoglobin (HbA1c) {mean deviation (MD) = -0.33, 95% confidence interval (CI) (-0.59, -0.07), p = 0.01}, Insulin {standard mean deviation (SMD) = -0.48, 95% CI (-0.74, -0.22), p = 0.0003} and Homeostatic Model Assessment for Insulin Resistance (HOMA-IR){SMD = -1.36, 95% CI (-2.30, -0.41), p = 0.005} than placebo group. No statistically significant differences were found regarding fasting blood glucose (FBG) and body mass index (BMI) {SMD = -0.39, 95% CI (-0.83, 0.05), p = 0.08}, {SMD = -0.40, 95% CI (-1.07, 0.27), p = 0.25}, respectively. Subgroup analyses, grouped by intervention times, showed that six to eight weeks of intervention improved HbA1c compared to the control group (p < 0.05), both six to eight weeks and 12-24 weeks had a better intervention effect on Insulin, and HOMA-IR (p < 0.05).In contrast, there was no statistically significant variation in the length between FBG and BMI regarding duration.

**Conclusion:**

This meta-analysis found probiotics at different intervention times play a positive role in modulating glucose in T2DM, specifically for HbA1c in six to eight weeks, Insulin and HOMA-IR in six to eight weeks, and 12-24 weeks. To confirm our findings, further excellent large-sample research is still required.

**Systematic review registration:**

https://www.crd.york.ac.uk/prospero, identifier CRD42023483325.

## 1 Introduction

Patients with type 2 diabetes mellitus are often associated with obesity and lack of physical activity (1). The prevalence of T2DM has been on the rise globally. According to estimates, the prevalence of T2DM constituted over 80% of diabetes cases across all 204 nations and territories (2). It is reported that 536.6 million people have diabetes in 2021 and increased to 783.2 million by 2045, among them, with China taking the top spot with 140.9 million in 2021, and the global healthcare costs of diabetes were $966 billion in 2021 (3). Therefore, more effective interventions are needed to address the increasing prevalence and the severe status (4).

Although there has been no cure for T2DM to date, effective ways are still available to delay and control its glucose levels, weight, and associated complications through lifestyle modifications and medication treatment (5). A healthy lifestyle was highly vital, including regular exercise, appropriate dietary habits, sound sleep, etc. (6). Prior network meta-analysis involved 471,038 patients and evaluated 13 different hypoglycemic drugs and their benefits (7). However, hypoglycemic drugs have adverse effects, including the increased risk of ketoacidosis and gastrointestinal adverse events, etc. (8). Therefore, relatively safe and effective methods are needed to be the option for glucose-lowering. Recently, Modifying the host microbiota has been suggested as an approach to cure or prevent various medical conditions (9), including non-alcoholic fatty liver disease (10), helicobacter pylori infection (11), etc. Moreover, emerging data indicates that the makeup of gut microbiota is a crucial pathophysiological component associated with T2DM (12). Furthermore, impaired host glycemic regulation might be related to unbalanced gut microbiota (13). Fortunately, current therapies can adjust gut microbiota in T2DM, for example, by adopting fecal microbiota transplantation, taking dietary fiber, exercising, and using probiotics (14).

Probiotics emerged in 1974 and have conceptually developed into the current standard definition of live microorganisms beneficial to health when ingested in sufficient amounts reported in clinical trials and animal experiments (15, 16). On the one hand, probiotics positively relieve patients’ gastrointestinal symptoms and increase medication tolerance (17). On the other hand, Hsieh et al. (18) discovered that streptozotocin (STZ) may protect β-cells, stabilize glucose levels, and reduce inflammation in diabetic animal models. However, a systematic review of 33 clinical trials investigating the probiotics’ effectiveness showed inconsistent results in glycemic control; not all glucose-related parameters were improved (19). Although the published systematic review and meta-analysis(SMRA) provided valuable and insightful information, it also revealed conflicting results of the glucose-control effect. There are mainly two reasons leading to the inconsistent results. Firstly, almost every single SMRA included at least two RCTs’ contents associated with synbiotics (20–26), which may lead to a confounding conclusion (27) owing to the definitions and mechanisms between probiotics and synbiotics being different (28). Besides, some studies reported that different intervention durations are effective for glucose-lowering, ranging from eight weeks (20, 21, 24) to 12 weeks (22, 23, 25, 26). However, no consensus exists on the optimal time for clinical staff and patients. Therefore, we need to consider these two factors and make appropriate adjustments to evaluate the published RCTs further to provide more precise evidence.

In this meta-analysis, we intended to examine the evidence regarding glycemic management in patients with T2DM using probiotics and further investigate the association between different intervention times on probiotics and glucose-lowering effect.

## 2 Methods

### 2.1 Protocol

We adhered to the Preferred Reporting Items for Systematic Reviews and Meta-Analysis (PRISMA) statement (**Supplementary Table 1**) (29). This study is registered in the International Prospective Register of Systematic Reviews (PROSPERO), registered number(CRD42023483325).

### 2.2 Search strategy

Four databases, PubMed, Embase, Web of Science, and Cochrane Library were searched from the inception until November 16, 2023, without any language limitations. We conducted the literature using a combination of medical subject headings and free terms to ensure a thorough retrieval of relevant papers. The search terms were:(type 2 diabetes mellitus OR diabetes mellitus, noninsulin-dependent OR diabetes mellitus) AND (probiotics OR lactobacillus OR bifidobacterial OR saccharomyces) AND (glycemic control OR control glycemic OR blood glucose control OR control blood glucose) AND (randomized controlled trial* OR randomized OR placebo). In addition, we adjusted the search strategy to meet the requirements of the four databases mentioned above. The complete strategy of each database is shown in **Supplementary Table 2**.

### 2.3 Inclusion and exclusion criteria

The following were the inclusion criteria: (1) participants were confirmed with a definite diagnosis of T2DM, which could be presented in two ways, namely, one was to explicitly mention the diagnostic criteria for T2DM and cite the literature in the article, and the other wrote explicit clinical diagnostic criteria in the text, (2) included study participants age were older than 18 years, (3) the intervention group received probiotics, and the control group received conventional treatment or placebo, (4) at least two of the below indicators were included in the literature: FBG, HbA1c, Insulin, HOMA-IR, BMI, (5) randomized controlled trials. The following were the exclusion standards: (1) there are apparent errors of data, or the data was incomplete to be merged, (2) reviews, conference papers, academic papers, and animal experiments were withdrawn, (3) non-Chinese or non-English literature, (4) included study’s full text cannot be downloaded.

### 2.4 Data extraction

Two authors independently screened the included studies. Based on the specified inclusion and exclusion criteria, we read the studies’ title, abstract, and full text, using Microsoft Excel to record the studies’ baseline information, which included author, publishing year, country, sample size, mean age, specific intervention of both probiotics and placebo group, intervention duration, outcome measurements.

### 2.5 Risk of bias assessment

Two reviewers independently utilized the Cochrane Handbook (30). The evaluation covered seven aspects: (1) the generation of random sequences, (2) the allocation and concealment of random programs, (3) the blinding of subjects and interventions, (4) the blinding of outcome evaluators, (5) the integrity of data indicators, (6) the possibility of selective reporting of research results, (7) other sources of bias. Based on the evaluation indicators, two evaluators evaluated from low and high risk of bias to unclear assessment, respectively. If there was a disagreement of opinion, a third evaluator would participate in the discussion and eventually reached a consensus.

### 2.6 Statistical analysis

We used Review Manager (RevMan) 5 (version 5.4) software to perform Meta-analysis. MD measured continuous variables with identical measurement units, and variables with varied measurement units were calculated by SMD and with 95% CI. A heterogeneity test was performed based on the research results, evaluated by I square statistic (I^2^). When p > 0.01 and I² < 50%, the results were considered homogeneous using a fixed effect model. When p < 0.01, I² ≥ 50%, the results were supposed to be heterogeneous using a random effect model. Subgroup analysis was performed based on intervention time. A p < 0.05 was considered statistically significant.

We used the final changes of mean and standard deviation values (SD) between the baseline and endpoint of the two groups for meta-analysis. If the last changes were not presented in the text, we would use the following three methods to process the data: (1) if the mean and SD before and after intervention were given, final changes were calculated with the formula (31). Specifically, the mean changes were equal to the after-intervention minus the before-intervention, and SD changes were computed using the formula: SD = SQRT (SD1^2^ + SD2^2^ - (2*R*SD1*SD2)) R = 0.5, (2) median and interquartile spacing are given in the text, and the mean and SD was calculated using formula (32, 33), then the computed value was converted to mean and SD according to the formula (31), (3) standard errors was given in the text, and SD was calculated based on a data calculator on the Cochrane website (34). Notably, all calculated values were retained to two decimal places using rounding.

## 3 Results

### 3.1 Included articles

Searching four databases, we screened 973 articles and imported them into Endnote software. **Supplementary Figure 1** depicts the literature selection. Among them, 365 studies were duplicated, and one was a retracted paper. Two authors read the title and abstract and excluded 554 studies independently. Then, two authors read 53 articles’ full text. Among them, 19 could not download the full text, 12 did not report clear diagnostic criteria for T2DM, six had less than two outcome indicators, four had more than two research design groups, two reported synbiotics, one was an animal experiment, and one had an incorrect value. Eventually, we included eight studies that met the inclusion criteria for quantitative analysis.

### 3.2 Study characteristics

We included eight RCTs (35–42) involving a total of 507 participants, with 252 in the probiotic group and 255 in the placebo group. In terms of geographical location, three were conducted in Iran (35, 36, 39), two in Ukraine (40, 41), and one each from Brazil (37), Malaysia (38), and Greece (11). In terms of published year, four studies were published before 2018 (35–38), and four studies were published after 2018 (39–42). The detailed information on the included studies is presented in **Tables 1**–**3**.

**Table 1:** Demographic characteristics of the included studies.

Study ID	Country	Sample size (n)		Age, years (Mean ± SD)	
		Probiotics	Control	Probiotics	Control
Asemi et al., 2013 (35)	Iran	27	27	50.51 ± 9.82	52.59 ± 7.14
Mazloom et al., 2013 (36)	Iran	16	18	55.4 ± 8	51.8 ± 10.2
Tonucci et al., 2017 (37)	Brazil	23	22	51.83 ± 6.64	50.95 ± 7.20
Firouzi et al., 2017 (38)	Malaysia	35	35	52.9 ± 9.2	54.2 ± 8.3
Razmpoosh et al., 2019 (39)	Iran	30	30	58.6 ± 6.5	61.3 ± 5.2
Kobyliak et al., 2020 (40)	Ukraine	28	26	56.29 ± 11.14	55.73 ± 8.76
Savytska et al., 2023 (41)	Ukraine	34	34	53.82 ± 9.58	56.93 ± 9.88
Zikou et al., 2023 (42)	Greece	46	45	64.54 ± 11.12	65.71 ± 10.82

**Table 2:** Findings and assessments of the included studies.

Study ID	Intervention		Times (week)	Outcome
	Probiotics	Control		
Asemi et al.2013 (35)	L.acidophilus(2×10^9^ CFU), L.casei(7×10^9^ CFU), L.rhamnosus(1.5×10^9^ CFU), L.bulgaricus(2×10^8^ CFU), Bifidobacterium breve(2×10^10^ CFU), B.longum(7×10^9^ CFU), Streptococcus thermophilus(1.5×10^9^ CFU)	placebo	8	FPG/HbA1c/HOMA-IR/Insulin
Mazloom et al.2013 (36)	L.acidophilus, L.bulgaricus, L.bifidum, and L. casei	placebo	6	FPG/HOMA-IR/Insulin
Tonucci et al.2017 (37)	10^9^CFUs of Lactobacillus acidophilus La-5, 10^9^ CFUs of 83 Bifidobacterium animals subsp and lactis BB-12	placebo	6	FPG/HbA1c/HOMA-IR/Insulin
Firouzi et al.2017 (38)	Lactobacillus, Firmicutes phyla, Bifidobacterium and Actinobacteria phyla	placebo	12	FPG/HbA1c/HOMA-IR/Insulin/BMI
Razmpoosh et al.2019 (39)	Lactobacillus acidophilus(2×10^9^ CFU), L.casei(7×10^9^ CFU), L.rhamnosus(1.5×10^9^ CFU), L.bulgaricus(2×10^8^ CFU), Bifidobacterium breve(3×10^10^ CFU), B.longum(7×10^9^ CFU), Streptococcus thermophilus(1.5×10^9^ CFU)	placebo	6	FPG/HOMA-IR/Insulin/BMI
Kobyliak et al.2020 (40)	Lactobacillus(1.0×10^9^ CFU/g), Bifidobacterium(1.0×10^9^ CFU/g), Lactococcus(1.0×10^8^ CFU/g), Propionibacterium(1.0×10^8^ CFU/g), Acetobacter(1.0×10^5^ CFU/g)	placebo	8	FPG/HbA1c/BMI
Savytska et al.2023 (41)	Lactobacillus+Lactococcus(6×10^10^ CFU/g), Bifidobacterium(1×10^10^/g), Propionibacterium(3×10^10^/g), Acetobacter (1×10^6^/g)	placebo	8	FPG/HbA1c
Zikou et al.2023 (42)	Lactobacillus acidophilus(1.75×10^9^ CFU), L.plantarum(0.5×10^9^ CFU), Bifidobacterium lactis(1.75×10^9^ CFU), Saccharomyces boulardii(1.5×10^9^ CFU)	placebo	24	FPG/HbA1c/BMI

**Table 3:** Findings and assessments of the included studies.

Study ID	Intervention		Times (week)	Delta after-before treatment
	Probiotics	Control		
Asemi et al., 2013 (35)	L.acidophilus(2×10^9^ CFU), L.casei(7×10^9^ CFU), L.rhamnosus(1.5×10^9^ CFU), L.bulgaricus(2×10^8^ CFU), Bifidobacterium breve(2×10^10^ CFU), B.longum(7×10^9^ CFU), Streptococcus thermophilus(1.5×10^9^ CFU)	placebo	8	FBG:-27.2±-27.35 HbA1c:-0.48 ± 0.22 HOMA-IR:-1.6±-0.34 Insulin:-0.27±-0.34
Mazloom et al., 2013 (36)	L.acidophilus, L.bulgaricus, L.bifidum, and L. casei	placebo	6	FBG:-12.71±-0.13 HOMA-IR:-0.84 ± 0.79 Insulin:-0.06 ± 0.12
Tonucci et al., 2017 (37)	10^9^CFUs of Lactobacillus acidophilus La-5, 10^9^ CFUs of 83 Bifidobacterium animals subsp and lactis BB-12	placebo	6	FBG:0.36±-0.1 HbA1c:-0.49 ± 0.3 HOMA-IR:-1 ± 1.81 Insulin:-0.45 ± 0.57
Firouzi et al., 2017 (38)	Lactobacillus, Firmicutes phyla, Bifidobacterium and Actinobacteria phyla	placebo	12	FBG:-0.4±-0.6 HbA1c:-0.16 ± 0.06 HOMA-IR:-1.3±-0.2 Insulin:-4.7±-0.5 BMI:-1.1 ± 0.1
Razmpoosh et al., 2019 (39)	Lactobacillus acidophilus(2×10^9^CFU), L.casei(7×10^9^ CFU), L.rhamnosus(1.5×10^9^ CFU), L.bulgaricus(2×10^8^ CFU), Bifidobacterium breve(3×10^10^ CFU), B.longum(7×10^9^ CFU), Streptococcus thermophilus(1.5×10^9^ CFU)	placebo	6	FBG:-13.4 ± 2.45 BMI:-0.2 ± 0
Kobyliak et al., 2020 (40)	Lactobacillus(1.0×10^9^ CFU/g), Bifidobacterium(1.0×10^9^ CFU/g), Lactococcus(1.0×10^8^ CFU/g), Propionibacterium(1.0×10^8^ CFU/g), Acetobacter(1.0×10^5^ CFU/g)	placebo	8	FBG:-0.85±-0.19 HbA1c:-0.48±-0.27 BMI:-0.4±-2.37
Savytska et al., 2023 (41)	LactobacillusLactococcus(6×10^10^CFU/g), Bifidobacterium(1×10^10^/g), Propionibacterium(3×10^10^/g), Acetobacter (1×10^6^/g)	placebo	8	FBG:2.08 ± 1.16 HbA1c:0.14 ± 0.14 BMI:0.11±-0.11
Zikou et al., 2023 (42)	Lactobacillus acidophilus(1.75×10^9^ CFU), L.plantarum(0.5×10^9^ CFU), Bifidobacterium lactis(1.75×10^9^CFU), Saccharomyces boulardii(1.5×10^9^ CFU)	placebo	24	FBG:-1.11 ± 0.12 HbA1c:-0.59±-0.04 BMI:-0.68±-1.29

### 3.3 Risk of bias

Two researchers individually employed the Cochrane bias risk assessment tool to evaluate the included eight studies (35–42)regarding seven aspects, which are shown in **Supplementary Figures 2A**, **B**. Specific information: (1) the generation of random sequences: seven trials with low risk of bias used computer generated random list, and one trials (35) only mentioned random without concrete process was judged as unclear risk, (2)allocation concealment: seven studies with low risk of bias used identical appearance and encoded container, and one trial (36) didn’t describe the allocation methods was evaluated as unclear risk, (3) blinding method for investigator and participants: six studies adopted blinding were judged as low risk of bias, and remaining two studies (35, 36) with an unclear risk for not mentioned the approach, (4) blinding method for outcome measurement: four studies (37, 38, 40, 41) were judged as low risk of bias, other four trials with an unclear risk for that aspects, (5) the integrity of the outcome: eight studies rated as low risk of bias for specifically reported the lost to follow-up and withdraw reasons, and two (38, 41) of which did the intention-to-treat analysis or per-protocol analysis, (6) all studies had no selective reporting of outcomes were judged as low risk of bias, (7) each study reported no additional biases were judged as low risk of bias.

## 4 Outcome measurement

### 4.1 Meta-analysis for FBG

Eight RCTs (35–42) reported the impact of probiotics on FBG. There were 252 participants in the probiotic group and 255 in the placebo group. **Supplementary Figure 3A** showed high heterogeneity in the two groups (I^2^ = 83%, p < 0.00001). Therefore, a random effect model was utilized. Meta-analysis revealed no statistically significant difference between the two groups comparison {SMD = -0.39, 95% CI (-0.83,0.05), p = 0.08}. In subgroup analysis (**Supplementary Figure 4A**), intervention time showed no statistical difference between the probiotic and control groups.

### 4.2 Meta-analysis for HbA1c

Six RCTs (8, 10, 11, 13–15) reported the impact of probiotics on HbA1c. There were 206 participants in the probiotic group and 207 in the placebo group. **Supplementary Figure 3B** showed a high heterogeneity in both groups (I^2^ = 64%, p = 0.02). Therefore, a random effect model was adopted. Meta-analysis showed that using probiotics had a considerably lower HbA1c value than the control group {MD = -0.33, 95% CI (-0.59, -0.07), p = 0.01}. In subgroup analysis (**Supplementary Figure 4B**), patients with T2DM taking probiotics that lasted for six to eight weeks had a considerably lower HbA1c than the control group {MD = -0.48, 95%CI(-0.85, -0.11), p = 0.01}.

### 4.3 Meta-analysis for insulin

Four RCTs (35–38) reported the impact of probiotics on Insulin. There were 114 participants in the probiotic group and 120 in the placebo group. **Supplementary Figure 3C** showed an excellent homogeneity in each group (I^2^ = 0%, p = 0.53). Therefore, a fixed effect model was adopted. Meta-analysis showed that using probiotics had a considerably lower Insulin level than the control group {SMD = -0.48, 95% CI (-0.74, -0.22), p = 0.0003}. In subgroup analysis (**Supplementary Figure 4C**), patients with T2DM taking probiotics lasted for six to eight weeks, and 12 to 24 weeks had a substantially lower Insulin level than the control group {SMD = -0.44, 95%CI(-0.79, -0.09), p = 0.01}, {SMD = -0.53, 95%CI(-0.93, 0.13), p = 0.009}, respectively.

### 4.4 Meta-analysis for HOMA-IR

Four RCTs (35–38) reported the impact of probiotics on HOMA-IR. There were 114 participants in the probiotic group and 120 in the placebo group. **Supplementary Figure 3D** showed a high heterogeneity in each group (I^2^ = 90%, p < 0.00001). Therefore, a random effect model was adopted. Meta-analysis showed that using probiotics had a substantially lower HOMA-IR level than the control group {SMD = -1.36, 95% CI (-2.30,-0.41), p = 0.005}. In addition, the HOMA-IR outcome was presented with mean and IQR failed to convert for computation (39). In subgroup analysis(**Supplementary Figure 4D**), patients with T2DM taking probiotics lasted for six to eight weeks and 12 to 24 weeks had a greatly lower HOMA-IR level than the control group {SMD = -0.68, 95%CI (-1.08, -0.27), p = 0.0010}, respectively.

### 4.5 Meta-analysis for BMI

Five RCTs (38–42) reported the impact of probiotics on BMI. There were 186 participants in the probiotic group and 188 in the placebo group. **Supplementary Figure 3E** showed high heterogeneity in each group (I^2^ = 90%, p < 0.00001). Therefore, a random effect model was adopted. Meta-analysis showed no statistical significance between the two group comparisons {SMD = -0.40, 95% CI (-1.07, 0.27), p = 0.25}. In subgroup analysis (**Supplementary Figure 4E**), intervention time showed no statistical difference between the probiotic and control groups.

### 4.6 Sensitivity analysis

Using leave-one-out methods to evaluate publication bias, we found stable results in FBG and Insulin. We found declined heterogeneity after one study was removed in HbA1c values (42) (I^2^ = 10%) (**Supplementary Figure 5A**), HOMA-IR values (35) (I^2^ = 0%) (**Supplementary Figure 5B**), and BMI values (38) (I^2^ = 8%) (**Supplementary Figure 5C**), respectively.

### 4.7 Publication bias

We included less than 10 papers, so we were unable to make a funnel plot to evaluate publication bias.

## 5 Discussion

Although accumulated evidence reported probiotics’ potential to reduce blood glucose in T2DM, findings have been inconsistent. Moreover, less research focused on the relationship between different probiotic intervention times and glucose-lowering effects. This meta-analysis included eight RCTs with 507 participants, indicating that probiotics play a positive role in controlling glycemic parameters, and their impact is significantly associated with intervention time. The results demonstrated that probiotic intervention may modulate HbA1c, Insulin, and HOMA-IR levels compared to the control group. However, although FBG and BMI levels declined, there was no statistical difference. Additionally, subgroup analysis showed that HbA1c can be reduced after six to eight weeks of probiotics intervention, and both six to eight weeks and 12 to 24 weeks of probiotics intervention can decrease Insulin and HOMA-IR levels. Herein, we further demonstrated the beneficial glucose-lowering effect of probiotics and revealed the positive time-related relationship between probiotics and the glucose-lowering effect (25).

### 5.1 FBG

Our study indicated a lack of significant improvement in FBG levels after using probiotics, which is similar to the two previous studies (43, 44). However, a survey of 17 RCTs with 1,009 participants used probiotics with decreased FBG levels, which is inconsistent with our study, which resulted from the inclusion of three studies on metformin therapy and two synbiotic-related studies (45). A recommendation to improve the probiotic meta-analysis suggested that only probiotic-related studies should be included because the definitions of prebiotics, synbiotics, and probiotics are distinct; if all are included, it will lead to inaccurate results (25). In addition, subgroup analysis showed that FBG level was changed by the intervention time but with no statistical difference, which complies with a previous study with a duration of less than eight weeks (19). However, another study of 37 RCTs with 2,503 participants showed that FBG levels continued to fall from two to six months (24). Furthermore, another study showed that short-term interventions (< 12 weeks) resulted in a more significant decrease in FBG compared to long-term interventions (>12 weeks), but this study included the prediabetes population. The inconsistency between our research and those mentioned above may account for the limited number of papers included and varied populations. Moreover, Quigley et al. (46) suggested that probiotics take time to adjust induced shifts in the gut microbiome composition. Likewise, our results indicated that probiotics’ glucose-lowering effect is time-related, but the exact mechanism remains unclear and needs further exploration.

### 5.2 HbA1c

This meta-analysis indicated that probiotics had the potential to reduce HbA1c, which is similar to the results of Xu et al. (47) And Zarezadeh et al. (48). Besides, animal experiments showed that lipopolysaccharide levels could be significantly elevated in patients with T2DM, and it enters the circulation to induce inflammation, destroying the integrity of the intestinal tract and affecting glucose metabolism, mainly reflected in the elevation of HbA1c (49). Probiotics can significantly reduce lipopolysaccharide levels, alleviate endoplasmic reticulum stress, and improve insulin sensitivity (50). However, previous studies reached contradictory results compared to ours. For instance, one study of 28 RCTs with 1,947 subjects showed an unstable result due to limited reports on HbA1c (21). In another study of 13 RCTs with 818 subjects, only seven articles reported HbA1c, which may result in discrepancies due to the use of varied probiotic strains (22). Besides, a study of 31 RCTs with 5,219 subjects also showed no positive effect on HbA1c (51). Therefore, we hypothesize that the included number is not proportional to the final results. Besides, subgroup analysis showed that six to eight weeks of intervention decreased HbA1c levels, which aligns with the previous study (26). Additionally, some studies only included more than 12 weeks for analysis but with no statistical effect (52). Existing knowledge suggests that HbA1c reflects glucose control status over the past two to three months, and it can predict microvascular complications (53), indicating short-term intervention may better reflect glucose management. In addition, although HbA1c can reflect the average glucose level, it does not accurately reflect the fluctuation of glucose (54). Therefore, the effect of fluctuation in glucose after prolonged intervention time of probiotics can be explored in the future.

### 5.3 Insulin

This meta-analysis suggested that probiotics are associated with decreased insulin levels which is consistent with previous studies (26). The results indicated that probiotics can assist in reducing insulin levels in patients with T2DM. Serum insulin can assess the secretory function of pancreatic β-cells (55). In addition, insulin levels are associated with an increase in short-chain fatty acids(SCFAs), and its decrease may increase insulin levels and improve insulin resistance (56). Besides, animal experiments have shown that SCFAs play a positive role in the intestinal flora of T2D mice (43). However, there is an opposite conclusion (39), probably because it included synbiotics for analysis. Furthermore, Ye et al. (57) evaluated gut microbial modulators, including probiotics, prebiotics, and synbiotics, indicating that the use of the latter two can increase insulin levels. This result further suggested that the three should not be compared together due to conceptual differences to avoid confounding results. In addition, subgroup analysis showed that long-term and short-term probiotic interventions resulted in decreased insulin levels, which aligns with a previous study (24). However, Liang et al. (22) showed that the insulin levels have nothing to do with the different durations, possibly due to each of the 11 studies containing different probiotic strains. In comparison, there is no consensus on whether varied strains will impact the final results. However, the intestinal microbiota should be balanced, and probiotics function well (58). Consistent probiotic strains or the same number of strains could be included to analyze the effect (25).

### 5.4 HOMA-IR

This study suggested that probiotics are associated with decreased HOMA-IR levels, which conformed to the previous research (26, 45). However, one study with 39 trials of 3,517 participants showed no significant HOMA-IR improvements due to evident heterogeneity (58). Consumption of probiotics affects the gut microbiota composition, shrinks the intestinal epithelium, and suppresses the immune response, ultimately increasing insulin sensitivity (59, 60). Our subgroup analysis found that six to eight weeks and 12-24 weeks of duration can modulate HOMA-IR, which is similar to previous meta-analysis results (26, 58). In contrast to our findings, a study showed that more than eight weeks of duration can modulate HOMA-IR (24). Insulin resistance refers to the body’s compensatory secretions of more insulin to maintain blood glucose, leading to decreased efficiency of glucose uptake and utilizing insulin, eventually resulting in hyperinsulinemia to maintain stable blood glucose levels (61). Besides, HOMA-IR can reflect insulin resistance degree, which is positively significant in glucose management. Notably, in our subgroup analysis, we both analyzed the effect of short-term and long-term probiotics with positive results, excluding the other index confounding factors, so we also attained more precise results.

### 5.5 BMI

This meta-analysis demonstrated that the difference in BMI levels has no statistical significance compared to the control group. Similarly, the previous meta-analysis also showed the same result (62). However, a meta-analysis of 33 trials investigating probiotics’ effect on overweight and obesity found that BMI levels are decreased (63). Thus, we analyzed why the varied results may lie in the limited included papers because our study only included five papers that reported BMI values. Nevertheless, prior research has demonstrated the detrimental effects of obesity on the gut microbiome-bile acid metabolism in models of both diet-induced obesity and hereditary obesity (64). Thus, microbiota modulation could be a non-invasive approach to treating metabolic disorders, especially obesity (65). In addition, our subgroup analysis showed that both short-term and long-term interventions have no statistical significance on BMI. In contrast to our findings, BMI was significantly modified in participants with metabolic syndrome with ≥ 12 weeks duration (66). A trial lasting eight weeks or more showed a more significant decrease in BMI (67). The different results may contribute to the inconsistent sample size (68). Although the difference may be associated with the probiotic strains and duration, the specified mechanism should be substantially explored.

### 5.6 limitations

This study has limitations for improvement in the future. First, we did not search gray literature, which may lead to selection bias. Second, we only included probiotics-related articles, which may lead to a limited number of articles, but this further clarified the effect of the intervention and avoided interfering factors. Thirdly, we only made subgroup analyses on duration, however, which was consistent with the aim of the study. Fourthly, because the values of Insulin and HOMA-IR were described as M ± IQR, the standard deviation could not be calculated, which may affect the results. Lastly, we did not create a funnel plot because the paper number was less than 10, which may cause publication bias.

## 6 Conclusions

This meta-analysis found significant differences in glycemic control in T2DM between intervention times. Probiotic interventions may positively impact HbA1c, Insulin, and HOMA-IR, where short-term intervention reduced HbA1c, and both short-term and long-term intervention reduced Insulin and HOMA-IR. We require additional high-quality, large-scale investigations to confirm our results.

## Data availability statement

The original contributions presented in the study are included in the article/**Supplementary Material**. Further inquiries can be directed to the corresponding authors.

## Author contributions

XW: Conceptualization, Data curation, Methodology, Software, Writing – original draft, Writing – review & editing. LC: Formal analysis, Supervision, Writing – review & editing. CZ: Formal analysis, Supervision, Writing – review & editing. QS: Writing – review & editing. LZ: Investigation, Methodology, Data curation, Writing – review & editing. SZ: Investigation, Methodology, Data curation, Writing – review & editing. ZL: Investigation, Methodology, Data curation, Writing – review & editing. YL: Investigation, Data curation, Writing – review & editing.
