# Supplementary material for: Effect of probiotics at different intervention time on glycemic control in patients with type 2 diabetes mellitus: a systematic review and meta-analysis
Source: Front Endocrinol (Lausanne). 2024 Jul 24;15:1392306. doi: 10.3389/fendo.2024.1392306 (PMC11303337; doi:10.3389/fendo.2024.1392306)
Supplement: Supplementary file 1 [file DataSheet_1.docx]

***Supplementary Material***

**Effect of probiotics at different intervention time on glycemic control in patients with type 2 diabetes mellitus: a systematic review and meta-analysis**

Xinghui Wang, Lu Chen^*^, Chunling Zhang^*^, Qing Shi, Lei Zhu, Sisi Zhao, Zhiqin Luo, Yirun Long

* Correspondence:

Lu Chen [25321331@qq.com](mailto:25321331@qq.com) Chunling Zhang 1277319952@qq.com

**Supplementary Figures**


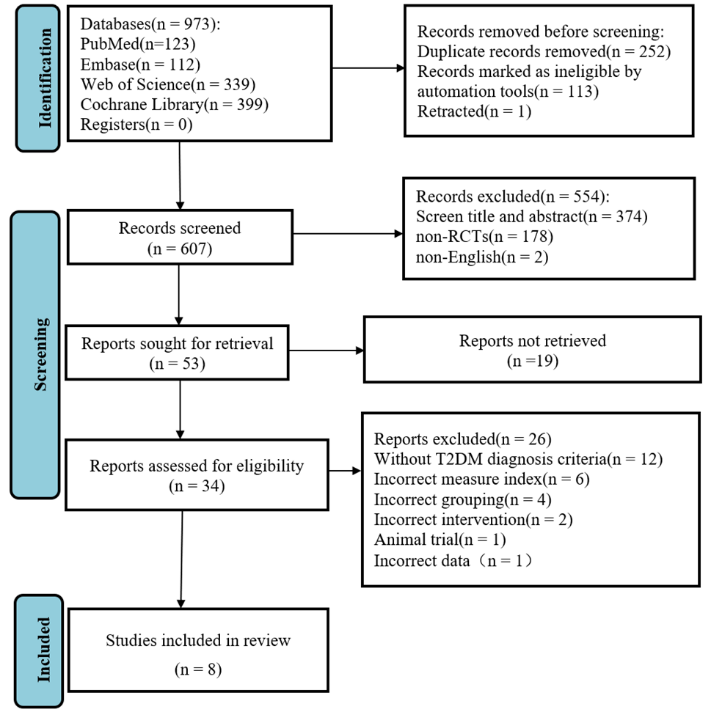


**Supplementary Figure 1.** Study selection flowgram through the PRISMA 2020.

**A**

**
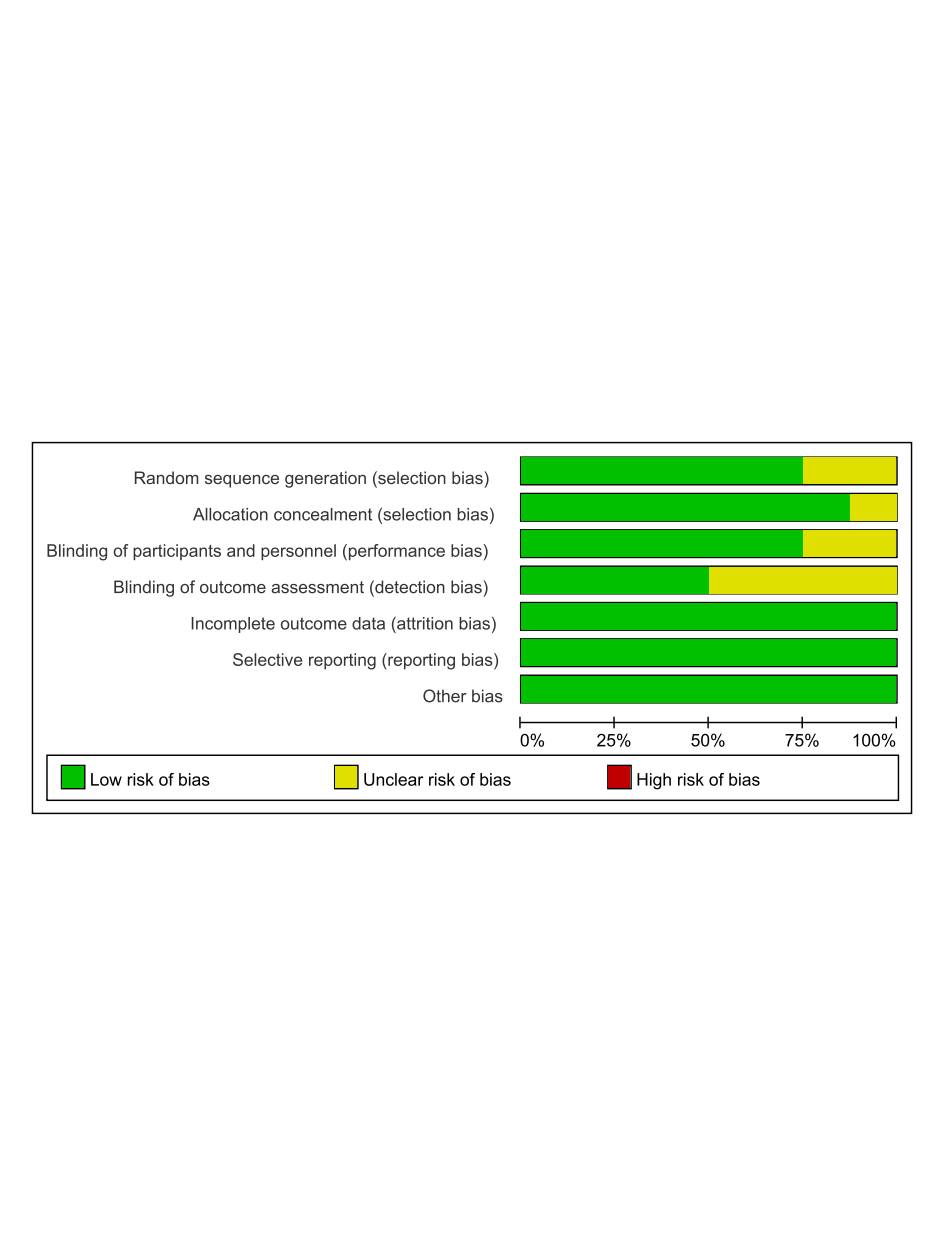
**

**B**

**
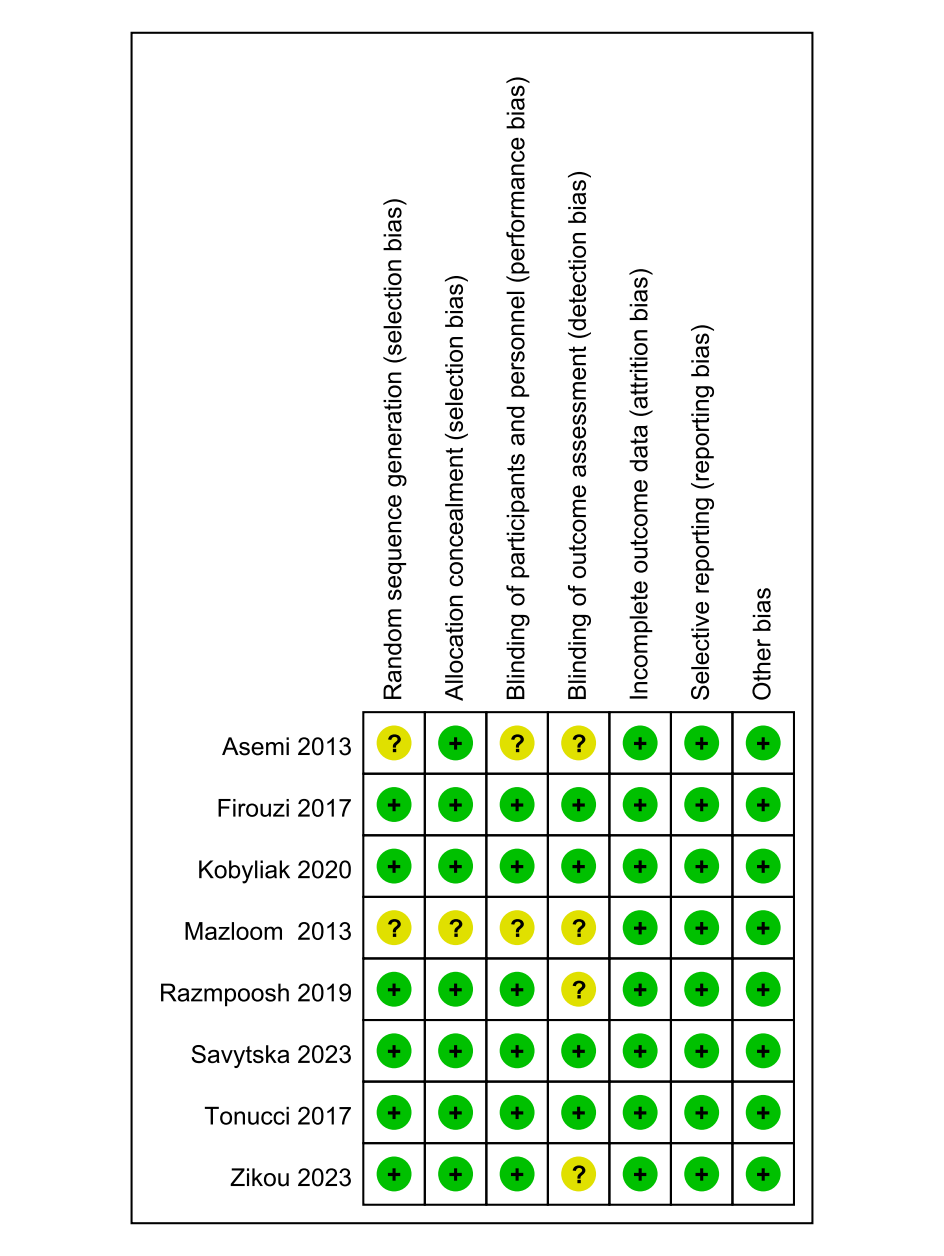
**

**Supplementary Figure 2.** Risk of bias assessment of included randomized controlled trials.

Risk of bias graph**(A)**.

Risk of bias summary**(B)**.

**A**
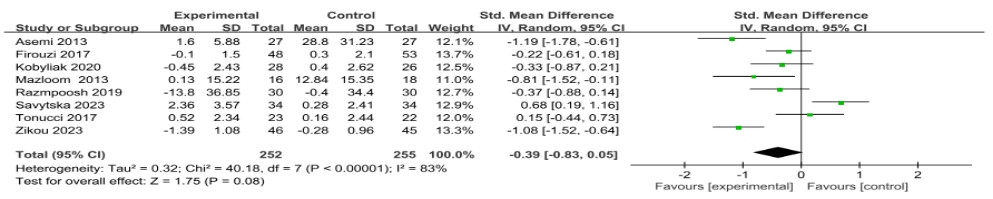
**B**
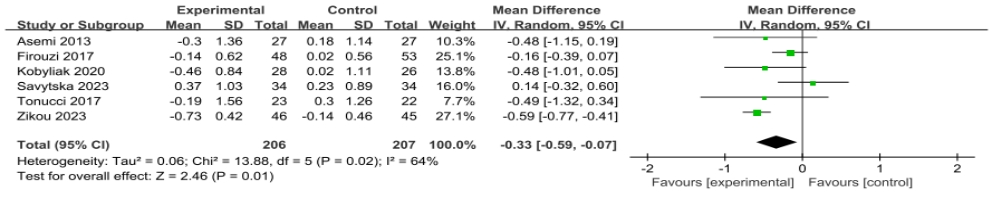


**C**
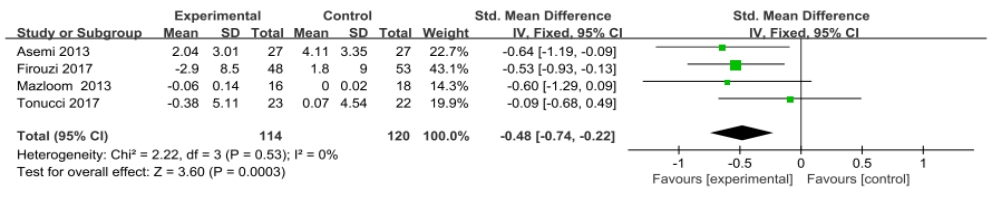
**D**
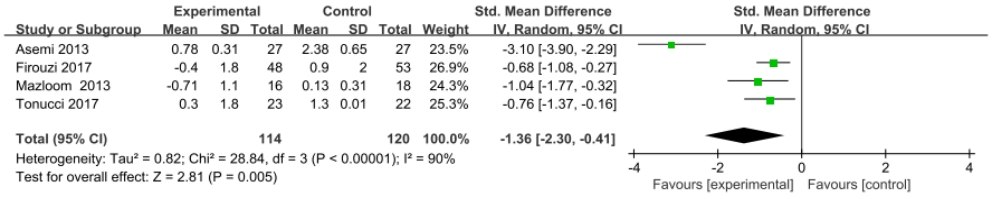
**E**
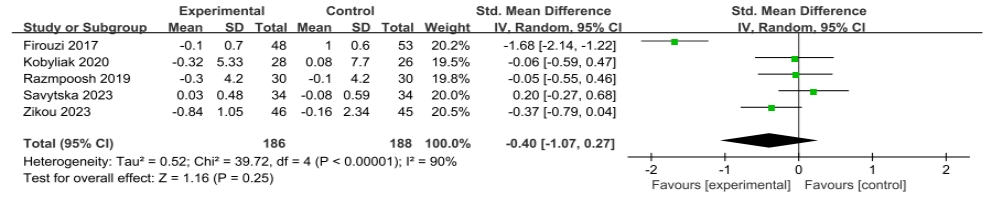
 **Supplementary Figure 3.** Forest plot of probiotics for five indicators.

Forest plot of probiotics on FBG level**(A)**.

Forest plot of probiotics on HbA1c level**(B)**.

Forest plot of probiotics on Insulin level**(C)**.

Forest plot of probiotics on HOMA-IR level**(D)**.

Forest plot of probiotics on BMI level**(E)**.

**A**


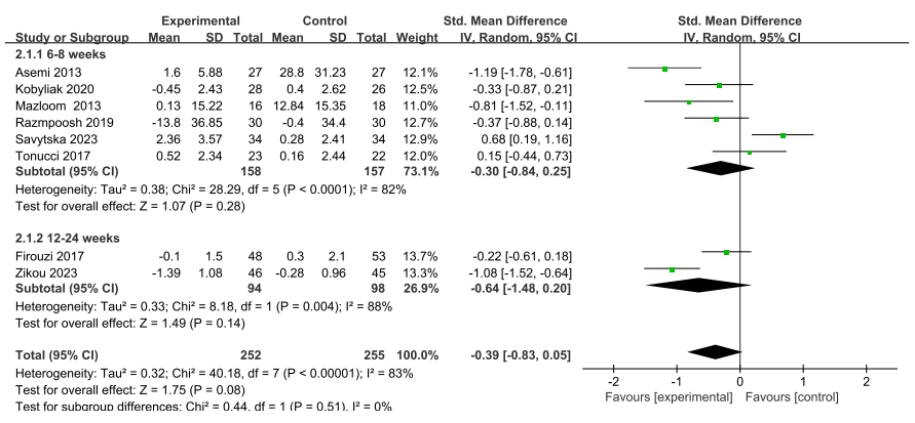


**B**


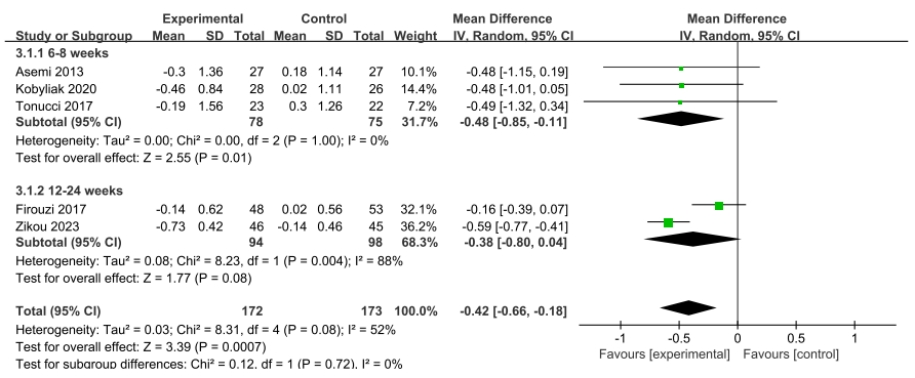


**C**


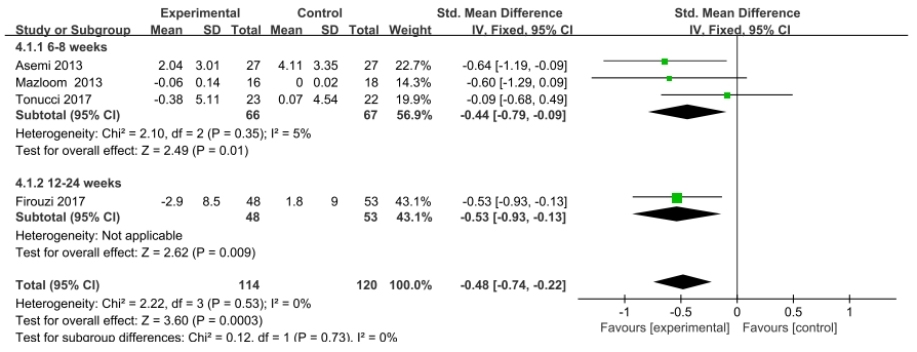


**D**


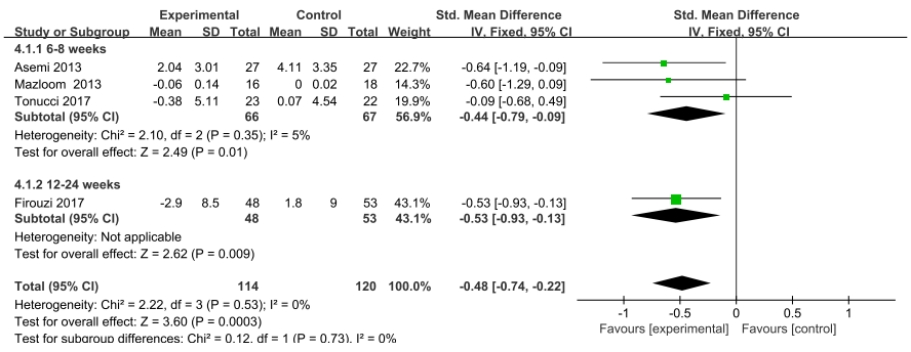


**E**


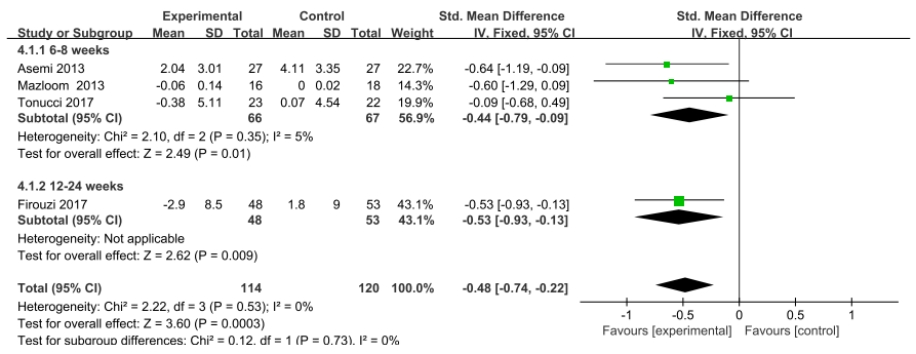


**Supplementary Figure 4.** Subgroup analysis of different probiotic intervention times on five indicators.

Subgroup analysis of different probiotic intervention times on FBG level**(A)**.

Subgroup analysis of different probiotic intervention times on HbA1C level**(B)**.

Subgroup analysis of different probiotic intervention times on HOMA-IR level**(C)**.

Subgroup analysis of different probiotic intervention times on Insulin level**(D)**.

Subgroup analysis of different probiotic intervention times on BMI level**(E)**.

**A**

**
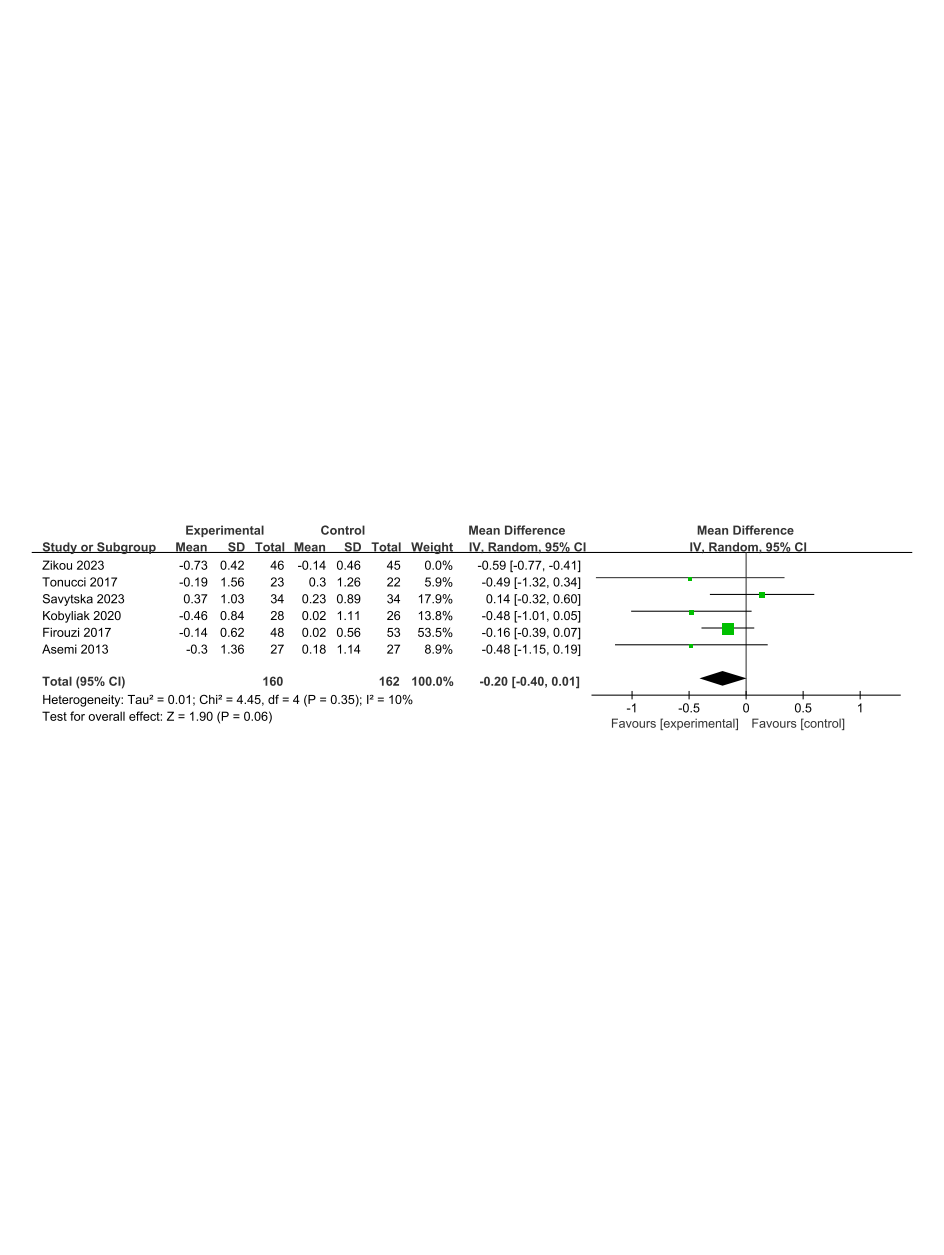
**

**B**

**
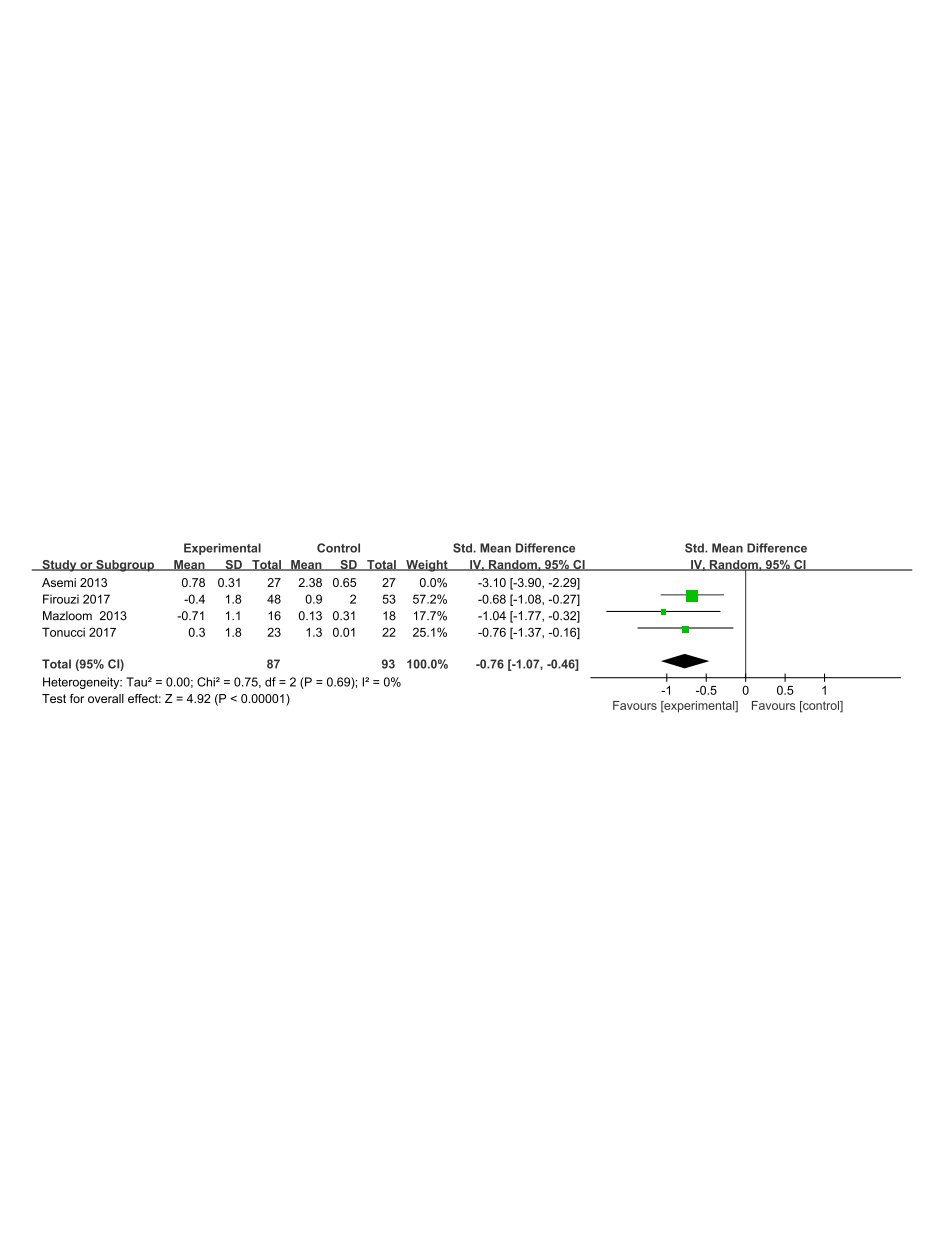
**

**C**

**
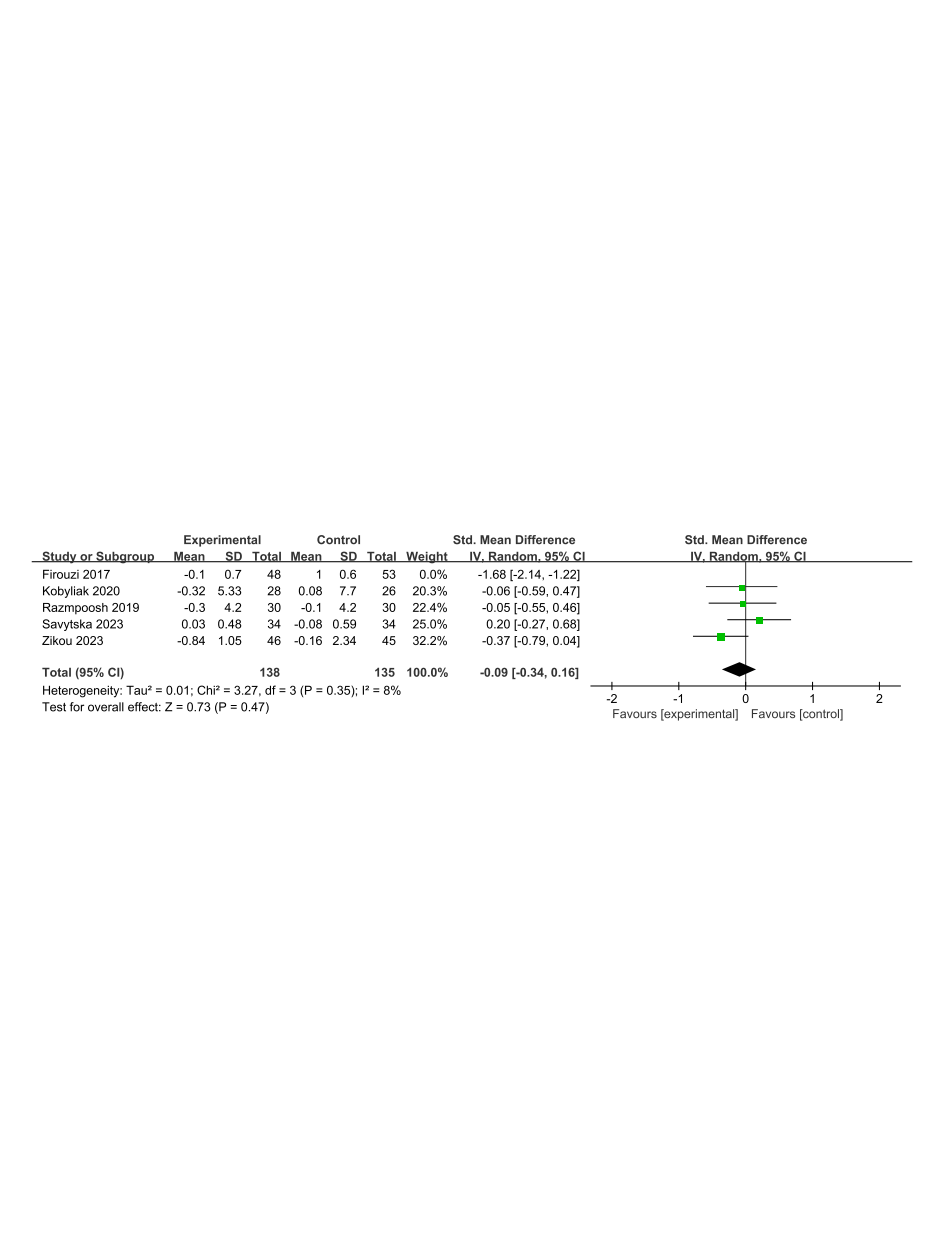
**

**Supplementary Figure 5.** Forest plot of sensitivity analysis for three indicators.

Forest plot of sensitivity analysis for HbA1c level**(A)**.

Forest plot of sensitivity analysis for HOMA-IR level**(B)**.

Forest plot of sensitivity analysis for BMI level**(C)**.
