# Supplementary material for: Effect of probiotics at different intervention time on glycemic control in patients with type 2 diabetes mellitus: a systematic review and meta-analysis
Source: Front Endocrinol (Lausanne). 2024 Jul 24;15:1392306. doi: 10.3389/fendo.2024.1392306 (PMC11303337; doi:10.3389/fendo.2024.1392306)
Supplement: Supplementary file 3 [file Table_2.pdf]

Supplementary Material

Effect of probiotics at different intervention time on glycemic control in patients with type 2 diabetes mellitus: a systematic review and meta-analysis

Xinghui Wang, Lu Chen*, Chunling Zhang*, Qing Shi, Lei Zhu, Sisi Zhao, Zhiqin Luo, Yirun Long

*** Correspondence: Lu Chen 25321331@qq.com Chunling Zhang 1277319952@qq.com**

Supplementary Tables

Supplementary Table 2. Search strategy. (from inception to November 16, 2023).

PubMed

#1	((((((((((((((((((((((((((((("Diabetes Mellitus, Type 2"[Mesh]) OR (Diabetes Mellitus, Noninsulin-Dependent[Title/Abstract])) OR (Diabetes Mellitus, Ketosis-Resistant[Title/Abstract])) OR (Diabetes Mellitus, Ketosis Resistant[Title/Abstract])) OR (Ketosis-Resistant Diabetes Mellitus[Title/Abstract])) OR (Diabetes Mellitus, Non Insulin Dependent[Title/Abstract])) OR (Diabetes Mellitus, Non-Insulin-Dependent[Title/Abstract])) OR (Non-Insulin-Dependent Diabetes Mellitus[Title/Abstract])) OR (Diabetes Mellitus, Stable[Title/Abstract])) OR (Stable Diabetes Mellitus[Title/Abstract])) OR (Diabetes Mellitus, Type II[Title/Abstract])) OR (NIDDM[Title/Abstract])) OR (Diabetes Mellitus, Noninsulin Dependent[Title/Abstract])) OR (Diabetes Mellitus, Maturity-Onset[Title/Abstract])) OR (Diabetes Mellitus, Maturity Onset[Title/Abstract])) OR (Maturity-Onset Diabetes Mellitus[Title/Abstract])) OR (Maturity Onset Diabetes Mellitus[Title/Abstract])) OR (MODY[Title/Abstract])) OR (Diabetes Mellitus, Slow-Onset[Title/Abstract])) OR (Diabetes Mellitus, Slow Onse[Title/Abstract])) OR (Slow-Onset Diabetes Mellitus[Title/Abstract])) OR (Type 2 Diabetes Mellitus[Title/Abstract])) OR (Noninsulin-Dependent Diabetes Mellitus[Title/Abstract])) OR (Noninsulin Dependent Diabetes Mellitus[Title/Abstract])) OR (Maturity-Onset Diabetes[Title/Abstract])) OR (Diabetes, Maturity-Onset[Title/Abstract])) OR (Maturity Onset Diabetes[Title/Abstract])) OR (Type 2 Diabetes[Title/Abstract])) OR (Diabetes, Type 2[Title/Abstract])) OR (Diabetes Mellitus, Adult-Onset[Title/Abstract])) OR (Adult-Onset Diabetes Mellitus[Title/Abstract])) OR (Diabetes Mellitus, Adult Onset[Title/Abstract])	237,636
#2	(((((((("Probiotics"[Mesh]) OR (Probiotic[Title/Abstract])) OR (lactobacillus[Title/Abstract])) OR (bifidobacterial[Title/Abstract])) OR (Saccharomyces[Title/Abstract])) OR (Propionibacterium[Title/Abstract])) OR (Enterococcus[Title/Abstract])) OR (Streptococcus[Title/Abstract])) OR (Saccharomyces[Title/Abstract])) OR (Yogurt[Title/Abstract])	260,272
#3	"Glycemic Control"[MeSH Terms] OR "control glycemic"[Title/Abstract] OR "blood glucose control"[Title/Abstract] OR "control blood glucose"[Title/Abstract] OR "glucose control blood"[Title/Abstract] OR "fasting plasma glucose"[Title/Abstract] OR "fasting blood sugar"[Title/Abstract] OR "glycosylated hemoglobin a1c"[Title/Abstract] OR "HbA1c"[Title/Abstract] OR "HOMA-IR"[Title/Abstract] OR "homeostasis model assessmentinsulin resistance"[Title/Abstract] OR "body mass index"[Title/Abstract] OR "BMI"[Title/Abstract] OR "insulin"[Title/Abstract] OR "fasting plasma insulin"[Title/Abstract]	736,732
#4	((randomized controlled trial*[Title/Abstract]) OR (randomized[Title/Abstract])) OR (placebo[Title/Abstract])	808,515
#5	#1 AND #2 AND #3 AND #4	123

Embase

#1	'non insulin dependent diabetes mellitus'/exp	345,634
#2	('adult onset diabetes':ab,ti OR 'adult onset diabetes mellitus':ab,ti OR 'diabetes mellitus type 2':ab,ti OR 'diabetes mellitus type ii':ab,ti OR 'diabetes mellitus, maturity onset':ab,ti OR 'diabetes mellitus, non insulin dependent':ab,ti OR 'diabetes mellitus, non-insulin-dependent':ab,ti OR 'diabetes mellitus, type 2':ab,ti OR 'diabetes mellitus, type ii':ab,ti OR 'diabetes type 2':ab,ti OR 'diabetes type ii':ab,ti OR 'diabetes, adult onset':ab,ti OR 'dm 2':ab,ti OR 'insulin independent diabetes':ab,ti OR 'insulin independent diabetes mellitus':ab,ti OR 'ketosis resistant diabetes mellitus':ab,ti OR 'maturity onset diabetes':ab,ti OR 'maturity onset diabetes mellitus':ab,ti OR 'maturity onset diabetes of the young':ab,ti OR 'niddm':ab,ti OR niddm:ab,ti) AND 'non insulin dependent diabetes mellitus':ab,ti OR 'non insulin	287,575

	dependent diabetes':ab,ti OR 'non-insulin-dependent diabetes mellitus':ab,ti OR 'noninsulin dependent diabetes':ab,ti OR 'noninsulin dependent diabetes mellitus':ab,ti OR 't2dm':ab,ti OR 'type 2 diabetes':ab,ti OR 'type 2 diabetes mellitus':ab,ti OR 'type ii diabetes':ab,ti OR 'type ii diabetes mellitus':ab,ti OR 'non insulin dependent diabetes mellitus':ab,ti	
#3	'probiotic agent'/exp	54,184
#4	'probiotic':ab,ti OR 'probiotics':ab,ti OR 'probiotic agent':ab,ti OR lactobacillus:ab,ti OR bifidobacterial:ab,ti OR propionibacterium:ab,ti OR enterococcus:ab,ti OR streptococcus:ab,ti OR saccharomyces:ab,ti OR yogurt:ab,ti	302,995
#5	'glycemic control'/exp	70,110
#6	'glycemic control':ab,ti OR 'control glycemic':ab,ti OR 'blood glucose control':ab,ti OR 'control blood glucose':ab,ti OR 'glucose control blood':ab,ti OR 'fasting plasma glucose':ab,ti OR 'fasting blood sugar':ab,ti OR 'glycosylated hemoglobin a1c':ab,ti OR hba1c:ab,ti OR 'homa ir':ab,ti OR 'homeostasis model assessmentinsulin resistance':ab,ti OR 'body mass index':ab,ti OR bmi:ab,ti OR insulin:ab,ti OR 'fasting plasma insulin':ab,ti	1,166,902
#7	'randomized controlled trial'/exp	794,214
#8	'controlled trial, randomized':ab,ti OR 'randomised controlled study':ab,ti OR 'randomised controlled trial':ab,ti OR 'randomized controlled study':ab,ti OR 'trial, randomized controlled':ab,ti OR 'randomized controlled trial':ab,ti	198,409
#9	#1 OR #2	399,125
#10	#3 OR #4	316,977
#11	#5 OR #6	1,185,420
#12	#7 OR #8	835,981
#13	#9 AND #10 AND #11 AND #12	112

Web of Science

#1	(TS=(type 2 diabetes mellitus)) OR TS=(Diabetes Mellitus, Type 2 OR Diabetes Mellitus, Noninsulin-Dependent OR Diabetes Mellitus, Ketosis-Resistant OR Diabetes Mellitus, Ketosis Resistant OR Ketosis-Resistant Diabetes Mellitus OR Diabetes Mellitus, Non Insulin Dependent OR Diabetes Mellitus, Non-Insulin-Dependent OR Non-Insulin-Dependent Diabetes Mellitus OR Diabetes Mellitus, Stable OR Stable Diabetes Mellitus OR Diabetes Mellitus, Type II OR NIDDM OR Diabetes Mellitus, Noninsulin Dependent OR Diabetes Mellitus, Maturity-Onset OR Diabetes Mellitus, Maturity Onset OR Maturity-Onset Diabetes Mellitus OR Maturity Onset Diabetes Mellitus OR MODY OR Diabetes Mellitus, Slow-Onset OR Diabetes Mellitus, Slow Onse OR Slow-Onset Diabetes Mellitus OR Type 2 Diabetes Mellitus OR Noninsulin-Dependent Diabetes Mellitus OR Noninsulin Dependent Diabetes Mellitus OR Maturity-Onset Diabetes OR Diabetes, Maturity-Onset OR Maturity Onset Diabetes OR Type 2 Diabetes OR Diabetes, Type 2 OR Diabetes Mellitus, Adult-Onset OR Adult-Onset Diabetes Mellitus OR Diabetes Mellitus, Adult Onset)	492,807
#2	(TS=(Probiotics)) OR TS=(probiotic OR lactobacillus OR bifidobacterial OR Saccharomyces OR Propionibacterium OR Enterococcus OR Streptococcus OR Saccharomyces OR Yogurt)	813.180

#3	(TS=(Glycemic Control)) OR TS=(control glycemic OR blood glucose control OR control blood glucose OR glucose control blood OR fasting plasma glucose OR fasting blood sugar OR glycosylated hemoglobin a1c OR HbA1c OR HOMA-IR OR homeostasis model assessmentinsulin resistance OR body mass index OR BMI OR insulin OR fasting plasma insulin)	1,587,553
#4	(TS=(randomized controlled trial*)) OR TS=(randomized OR placebo)	1,496,893
#5	#1 AND #2 AND #3 AND #4	339

Cochrane Library

#1	MeSH descriptor: [Diabetes Mellitus, Type 2] explode all trees	23,475
#2	(Diabetes Mellitus, Type 2 OR Diabetes Mellitus, Noninsulin-Dependent OR Diabetes Mellitus, Ketosis-Resistant OR Diabetes Mellitus, Ketosis Resistant OR Ketosis-Resistant Diabetes Mellitus OR Diabetes Mellitus, Non Insulin Dependent OR Diabetes Mellitus, Non-Insulin-Dependent OR Non-Insulin-Dependent Diabetes Mellitus OR Diabetes Mellitus, Stable OR Stable Diabetes Mellitus OR Diabetes Mellitus, Type II OR NIDDM OR Diabetes Mellitus, Noninsulin Dependent OR Diabetes Mellitus, Maturity-Onset OR Diabetes Mellitus, Maturity Onset OR Maturity-Onset Diabetes Mellitus OR Maturity Onset Diabetes Mellitus OR MODY OR Diabetes Mellitus, Slow-Onset OR Diabetes Mellitus, Slow Onse OR Slow-Onset Diabetes Mellitus OR Type 2 Diabetes Mellitus OR Noninsulin-Dependent Diabetes Mellitus OR Noninsulin Dependent Diabetes Mellitus OR Maturity-Onset Diabetes OR Diabetes, Maturity-Onset OR Maturity Onset Diabetes OR Type 2 Diabetes OR Diabetes, Type 2 OR Diabetes Mellitus, Adult-Onset OR Adult-Onset Diabetes Mellitus OR Diabetes Mellitus, Adult Onset):ti,ab,kw (Word variations have been searched)	64,741
#3	MeSH descriptor: [Probiotics] explode all trees	3,102
#4	(Probiotics OR lactobacillus OR bifidobacterial OR Saccharomyces OR Propionibacterium OR Enterococcus OR Streptococcus OR Saccharomyces OR Yogurt):ti,ab,kw (Word variations have been searched)	18,792
#5	MeSH descriptor: [Glycemic Control] explode all trees	1,623
#6	(control glycemic OR blood glucose control OR control blood glucose OR glucose control blood OR fasting plasma glucose OR fasting blood sugar OR glycosylated hemoglobin a1c OR HbA1c OR HOMA-IR OR homeostasis model assessmentinsulin resistance OR body mass index OR BMI OR insulin OR fasting plasma insulin):ti,ab,kw (Word variations have been searched)	164,857
#7	MeSH descriptor: [Randomized Controlled Trial] explode all trees	25,732
#8	(randomized controlled trial* OR randomized OR placebo):ti,ab,kw (Word variations have been searched)	1,372,185
#9	#1 OR #2	64,742
#10	#3 OR #4	18,860
#11	#5 OR #6	164,857

#12	#7 OR #8	1,372,185
#13	#9 AND #10 AND #11 AND #12	399
